# NUPR1 promotes the proliferation and metastasis of oral squamous cell carcinoma cells by activating TFE3-dependent autophagy

**DOI:** 10.1038/s41392-022-00939-7

**Published:** 2022-04-25

**Authors:** Tengfei Fan, Xiaoning Wang, Sheng Zhang, Ping Deng, Yi Jiang, Yidan Liang, Sheng Jie, Qing Wang, Chuwen Li, Guocai Tian, Zhen Zhang, Zhenhu Ren, Bo Li, Yanrong Chen, Zhijing He, Yan Luo, Mingliang Chen, Hanjiang Wu, Zhengping Yu, Huifeng Pi, Zhou Zhou, Zhiyuan Zhang

**Affiliations:** 1 Department of Oral and Maxillofacial-Head Neck Oncology, Shanghai Ninth People’s Hospital, Shanghai Jiao Tong University School of Medicine; College of Stomatology, Shanghai Jiao Tong University; National Center for Stomatology; National Clinical Research Center for Oral Diseases; Shanghai Key Laboratory of Stomatology; Research Unit of Oral and Maxillofacial Regenerative Medicine, Chinese Academy of Medical Sciences, Shanghai, China; 2 Department of Oral and Maxillofacial Surgery, Zhang Zhiyuan Academician Workstation, Hainan Western Central Hospital, Shanghai Ninth People’s Hospital, Danzhou, Hainan China; 3 Department of Oral and Maxillofacial Surgery, The Second Xiangya Hospital of Central South University, Changsha, Hunan China; 4 Department of Oral Pathology, Shanghai Ninth People’s Hospital, Shanghai Jiao Tong University School of Medicine, Shanghai, China; 5 Department of Occupational Health, Third Military Medical University, Chongqing, China; 6 Department of Pathology, The Second Xiangya Hospital of Central South University, Changsha, Hunan China; 7 School of Medicine, Guangxi University, Nanning, Guangxi Zhuang Autonomous Region China; 8 Department of Oral and Maxillofacial Surgery, Affiliated Hospital of Guilin Medical University, Guilin, Guangxi Zhuang Autonomous Region China; 9 Institute of Pathology and Southwest Cancer Centre, Southwest Hospital, Third Military Medical University, Chongqing, China; 10 Department of Emergency Medicine, First Affiliated Hospital and Department of Environmental Medicine, School of Public Health, School of Medicine, Zhejiang University, Hangzhou, Zhejiang China

**Keywords:** Head and neck cancer, Head and neck cancer

## Abstract

Oral squamous cell carcinoma (OSCC) is the most common type of oral malignancy, and metastasis accounts for the poor prognosis of OSCC. Autophagy is considered to facilitate OSCC development by mitigating various cellular stresses; nevertheless, the mechanisms of autophagy in OSCC cell proliferation and metastasis remain unknown. In our study, high-sensitivity label-free quantitative proteomics analysis revealed nuclear protein 1 (NUPR1) as the most significantly upregulated protein in formalin-fixed paraffin-embedded tumour samples derived from OSCC patients with or without lymphatic metastasis. Moreover, NUPR1 is aberrantly expressed in the OSCC tissues and predicts low overall survival rates for OSCC patients. Notably, based on tandem mass tag-based quantitative proteomic analysis between stable *NUPR1* knockdown OSCC cells and scrambled control OSCC cells, we confirmed that NUPR1 maintained autophagic flux and lysosomal functions by directly increasing transcription factor E3 (TFE3) activity, which promoted OSCC cell proliferation and metastasis in vitro and in vivo. Collectively, our data revealed that the NUPR1–TFE3 axis is a critical regulator of the autophagic machinery in OSCC progression, and this study may provide a potential therapeutic target for the treatment of OSCC.

## Introduction

Oral squamous cell carcinoma (OSCC) is the most common form of head and neck neoplasms,^1,2^ and it has high prevalence rate, accounting for estimated 377,713 new global cases in 2020.^3,4^ Although surgical resection combined with chemotherapy and radiation has been applied in many cases, the overall 5-year survival rate of OSCC patients has not exceeded 50% in recent 20 years, without any obvious developments.^5,6^ In particular, metastasis is one of the deadliest aspects of oral cancer and can facilitate the dissemination of cancer cells to remote body locations, such as the lung, and the majority of metastatic OSCC patients die within one year.^7^ Thus, the underlying molecular mechanisms of OSCC proliferation and metastasis could provide vital information for the development of new therapeutic methods to prevent OSCC progression.

Autophagy is a dynamic recycling system that provides internal constituents and energy for cellular homoeostasis and renovation under stressful conditions such as the tumour microenvironment.^8,9^ Some researchers have linked autophagy to the inhibition of OSCC progression; however, other studies have demonstrated that autophagy is positively correlated with OSCC tumorigenesis and development. Tang *et al*. reported that OSCC had a high degree of autophagy activity, and dual expression of tumour autophagy protein 5 (ATG5) and Beclin-1 (BECN1) expression served as a poor prognostic indicator for OSCC.^10^ Moreover, in our previous report, we confirmed that OSCC tissues had higher autophagic activity than paracancerous tissues.^11^ Although upregulated autophagic activity may promote OSCC progression, the underlying mechanism of autophagy in OSCC is still unclear, which limits the targeting of autophagy as a treatment for OSCC.

Nuclear protein 1 (NUPR1) is strongly induced by several types of cellular stress and involves in the chromatin remodelling, cell cycle, and apoptosis.^12^ Importantly, NUPR1 has been demonstrated to facilitate the progression and promotes the metastasis of many malignancies, such as breast cancer, pancreatic adenocarcinoma, hepatocellular cancer and thyroid cancer.^12–14^ Additionally, emerging evidences have demonstrated that NUPR1 could be taken as an autophagic flux master. NUPR1 maintained autophagy process in lung cancer;^15^ moreover, *NUPR1* silencing decreased autophagy, leading to the accumulation of sequestosome 1 (SQSTM1) and facilitating multiple myeloma cells death.^16^ Our previous study reported that xenobiotic (e.g., Cd) exposure enhanced NUPR1 expression and initiated autophagy, which led to the OSCC progression.^17^ These reports suggest that NUPR1 may upregulate autophagic flux and drive OSCC metastasis, but the molecular mechanism remains poorly understood.

Transcription factor E3 (TFE3) has been identified as a powerful regulator that controls the autophagic flux-related genes expression in various cancers. Perera *et al*. reported that TFE3 enhanced lysosome biogenesis and function and augmented autophagy, which promoted the pathological process of pancreatic ductal adenocarcinoma.^18^ Moreover, our previous report and other study also confirmed that TFE3, which led to autophagic flux enhancement, was positively correlated with the development and poor prognosis of OSCC as well as breast cancer.^12,19^

In this study, high-sensitivity quantitative label-free quantitative proteomics analysis of formalin-fixed, paraffin-embedded (FFPE) tumour samples was combined with tandem mass tag (TMT) proteomic analysis of the *NUPR1* knockdown (KD) OSCC cell line to determine the possible mechanisms of autophagy involved in OSCC proliferation and metastasis. We revealed and confirmed that NUPR1 maintained autophagic flux by activating TFE3 transcription during OSCC progression. Therefore, understanding the NUPR1-TFE3-mediated autophagy process reveals potential new avenues for pharmacological therapies targeting OSCC progression.

## Results

### NUPR1 may be a vital protein involved in OSCC progression

We first performed high-sensitivity label-free quantitative proteomics analysis of FFPE tumour samples derived from OSCC patients with or without lymphatic metastasis (Fig. S1a). According to the quantification results with MaxQuant software, we identified and quantified 3021 proteins. The differentially expressed proteins (DEPs, *P* value <0.05 and a ratio-fold change ≥1.5 or ≤0.67) were clustered through unsupervised hierarchical clustering analysis, which showed the gathering characteristics and proteomic diversity between the two groups (Supplementary Fig. S1b). Compared with samples from OSCC patients without lymph node metastasis (LNM), those from patients with LNM exhibited 208 upregulated proteins and 165 downregulated proteins (Supplementary Fig. S1c). Furthermore, NUPR1 expression was notably upregulated in the LNM group compared with the non-LNM group (top five upregulated proteins) (Supplementary Table S1).

### NUPR1 was positively correlated with OSCC progression and adverse prognosis in patients

**Fig. 1:**
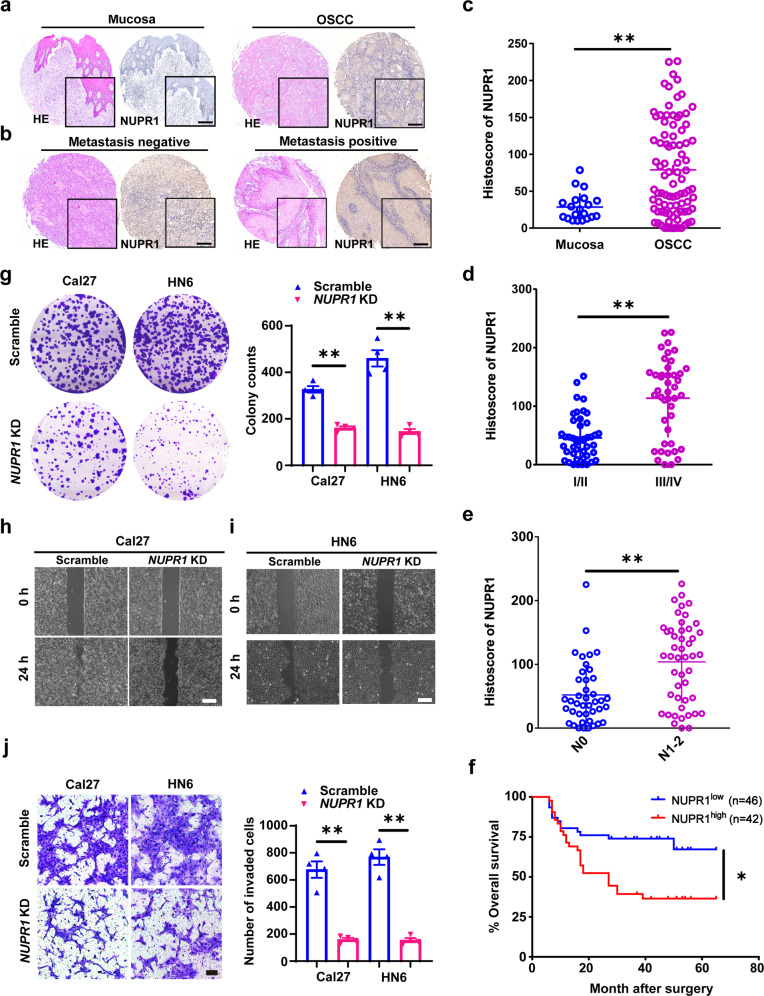
NUPR1 is correlated with OSCC progression. **a** Representative images of NUPR1 expression in normal oral mucosa (left) and OSCC (right) by IHC staining; scale bars = 100 μm. **b** Representative images of NUPR1 expression in non-LNM (left) and LNM (right) OSCC samples by IHC staining; scale bars = 100 μm. **c** NUPR1 expression was substantially upregulated in OSCC tissues (*n* = 88) compared with oral mucosa tissues (*n* = 20) using TMA analysis by IHC staining; **P < 0.01. **d** Histoscores of NUPR1 analysed by TMA in grade I/II tissues (*n* = 45) and grade III/IV tissues (*n* = 43); **P < 0.01. **e** Histoscore of NUPR1 analysed by TMA analysis in OSCC patients with LNM (N^+^, *n* = 47) and without LNM (N0, *n* = 41); **P < 0.01. **f** Kaplan–Meier survival curves of patients stratified according to high (*n* = 46) or low (*n* = 42) NUPR1 expression; *P < 0.05. **g** Representative images of colony formation and quantitative analysis results; *n* = 4. **h**, **i** Wound healing assays showed that *NUPR1* KD suppressed the migration of Cal27 or HN6 cells; scale bar = 100 μm. **j** The invasion results for *NUPR1* KD or scrambled Cal27 and HN6 cells at 48 h. Scale bar = 80 μm; magnification, 40×; *n* = 4; **P < 0.01 vs. the scrambled group

To further validate the proteomic findings in a larger patient cohort, the protein expression levels of NUPR1 were detected using our OSCC TMA with IHC. The results showed that NUPR1 expression was remarkably increased in OSCC tissues (*n* = 88) versus normal mucosa tissues (*n* = 20, Fig. 1a, c). We further explored the relationship between the expression level of NUPR1 (high or low) and several clinicopathological characteristics of OSCC patients (Supplementary Table S2). NUPR1 expression was categorised based on the median value according to immunohistochemistry (IHC) scores for 88 OSCC tissues. High expression of NUPR1 was statistically associated with pathological differentiation grade (low, *P* = 0.000), TNM stage (III/IV, *P* = 0.008) and lymphatic metastasis (N^+^, *P* = 0.000). The expression of NUPR1 positively correlated with TNM stage and lymphatic metastasis (n = 47, *P* < 0.05, Figs. 1b, d, e), suggesting that NUPR1 is positively related to OSCC metastasis. Moreover, according to Kaplan–Meier survival analysis and the log-rank test, the overall survival rate was lower for OSCC patients with high NUPR1 expression levels(*P* = 0.014, Fig. 1f).

### *NUPR1* knockdown (KD) inhibited the OSCC progression

**Fig. 2:**
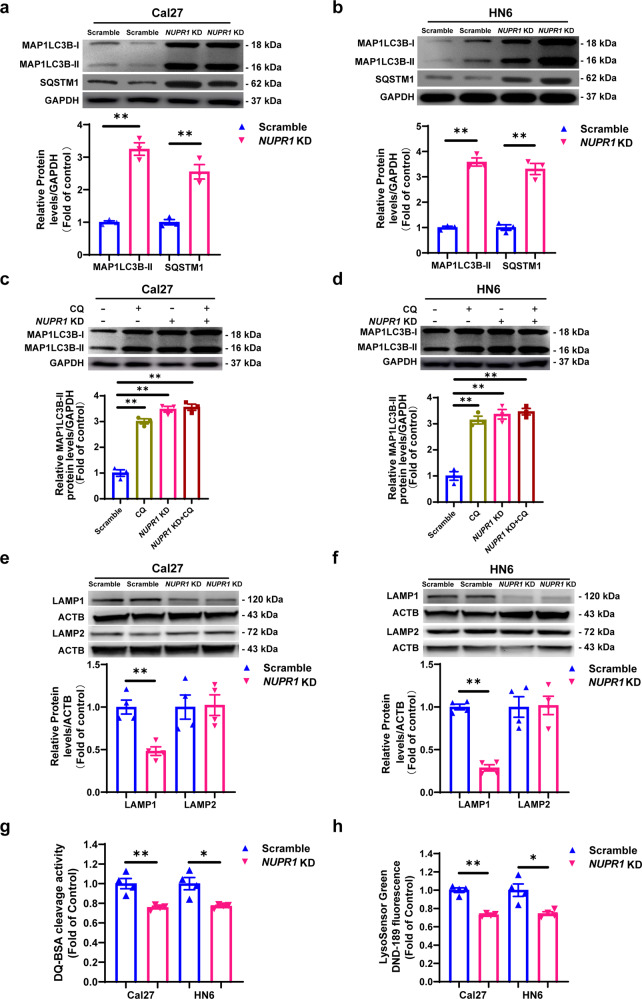
*NUPR1* KD restrained autophagic flux and lysosomal function in OSCC cells. **a**, **b** Immunoblotting analysis of MAP1LC3B-II and SQSTM1 levels in *NUPR1* KD or scrambled Cal27 and HN6 cells; *n* = 3. **c**, **d** Immunoblotting analysis of MAP1LC3B-II in *NUPR1* KD or scrambled Cal27 and HN6 cells in the absence or presence of CQ (10 μM) for 24 h; *n* = 3. **e**, **f** Immunoblotting analysis of LAMP1 and LAMP2 in *NUPR1* KD or scrambled Cal27 and HN6 cells; *n* = 4. **g** DQ-BSA staining fluorescence intensity in *NUPR1* KD or scrambled Cal27 and HN6 cells; *n* = 4. **h** LysoSensor DND-189 fluorescence intensity in *NUPR1* KD or scrambled Cal27 and HN6 cells. *n* = 4; *P < 0.05, **P < 0.01 vs. the scrambled group

To clarify the role of NUPR1 in OSCC progression, we firstly determined NUPR1 expression in six OSCC cell lines, and we confirmed that the expressions of NUPR1 in Cal27 and HN6 were higher than other cell lines (Supplementary Fig. S2a). Then Cal27 and HN6 cells were stably transfected with lentivirus-containing negative control or shRNA-*NUPR1* (Supplementary Fig. S2b). A colony formation assay revealed that *NUPR1* KD significantly decreased the colony formation efficiency of Cal27 and HN6 cells (Fig. 1g). In addition, wound healing and Matrigel-coated Transwell assays demonstrated that the migration and invasion abilities of *NUPR1*-depleted OSCC cells were markedly suppressed (Fig. 2h–j). Overall, *NUPR1* KD effectively suppressed the OSCC progression.

### Autophagy plays a crucial role in NUPR1-mediated OSCC progression

To clarify the underlying mechanism of NUPR1 in OSCC progression, we detected the protein profile changes between *NUPR1*-depleted Cal27 cells and scrambled control cells through TMT quantitative proteomic analysis. The levels of 27 proteins were significantly upregulated and the levels of 28 proteins were remarkably downregulated in the *NUPR1*-depleted OSCC group compared to the scrambled group (Supplementary Fig. S3a). Kyoto Encyclopedia of Genes and Genomes (KEGG) pathway analysis indicated that the autophagy pathway was markedly enriched in the *NUPR1* KD group compared to the scrambled group (Supplementary Fig. S3b). These results demonstrated that autophagy may play an essential role in NUPR1-mediated OSCC progression.

### *NUPR1* KD blocked autophagic flux in OSCC cells

Next, we further investigated whether *NUPR1* KD affected autophagic flux in OSCC cells. The MAP1LC3B-I protein plays an essential role in autophagy and is a well-admitted autophagy marker that normally resides in the cytosol, but upon induction of autophagy, it becomes lipidated and embedded in autophagosomal membranes (forming the MAP1LC3B-II isoform).^20^ Immunoblotting analysis showed that *NUPR1* KD increased the protein expression levels of MAP1LC3B-II (Fig. 2a, b). Additionally, SQSTM1 binds MAP1LC3B-II for cargo recruitment and degradation when it accumulates,^21^ was also apparently elevated in Cal27 and HN6 cells (Fig. 2a, b). Autophagy involves multiple steps, and autophagosome accumulation might occur on account of upregulated autophagy activity or decreased autophagosome turnover. *NUPR1* KD led to increased levels of MAP1LC3B-II and expression of SQSTM1, which seems to imply that *NUPR1* KD impaired OSCC cell autophagic flux. Chloroquine (CQ) inhibits lysosome activity and disturbs the fusion between autophagosomes and lysosomes to inhibit autophagy,^22^ however, the *NUPR1* KD-induced increase in MAP1LC3B-II expression was not affected by cotreatment with CQ (Fig. 2c, d). These results indicated that *NUPR1* KD inhibits autophagic flux in OSCC cells.

### *NUPR1* KD did not restrain autophagosome formation or maturation in OSCC cells

Since autophagy serves as a dynamic recycling system and has a multistep process, we investigated autophagy inhibition induced by *NUPR1* KD in the early or late stage. ATG5 conjugated with ATG12 and PIK3C3/VPS34 kinase activity is the requirement of phagophore formation in cells.^23^ We transfected Cal27 and HN6 cells with an *ATG5* shRNA plasmid in the presence or absence of *NUPR1* KD and observed a change in GFP-LC3 puncta, which represent phagophores. As shown in Supplementary Fig. S4a, b, *ATG5* shRNA attenuated the increase in GFP-LC3 puncta induced by *NUPR1* KD in Cal27 and HN6 cells. Similarly, *ATG5* KD reduced MAP1LC3B-II accumulation under *NUPR1* depletion conditions, indicating that *NUPR1* KD does not affect phagophore formation (Supplementary Fig. S4c, d). Polyubiquitinated protein aggregates, whose formation is mediated by SQSTM1, a cargo incorporation of autophagosomes, are crucial components of mature autophagosomes.^24^ We evaluated autophagosome maturation by assessing GFP-LC3 colocalization with SQSTM1. Compared with the scrambled group, the *NUPR1* KD group showed an augmented colocalization coefficient of GFP-LC3 and SQSTM1 in Cal27 and HN6 cells (Supplementary Fig. S4e, f). Together, these results indicated that *NUPR1* KD does not facilitate the cargo incorporation or maturation of autophagosomes in OSCC cells.

### *NUPR1* KD had no effect on autophagosome-lysosome fusion in OSCC cells

The fusion of autophagosomes with lysosomes is a fundamental process of autophagic degradation. As shown in Supplementary Fig. S5a–d, the ratio of colocalization between the autophagosomal marker GFP-LC3 and the lysosomal marker LAMP2 was not affected by *NUPR1* KD in Cal27 and HN6 cells. The RFP-GFP-LC3B kit can be used as an autophagy sensor to monitor fusion. GFP fluorescence is pH sensitive and is quenched in an acidic environment, but RFP fluorescence is not sensitive to changes in pH. Thus, yellow puncta indicate autophagosomes before fusion, and red puncta indicate complete autophagosome fusion.^25^ As shown in Supplementary Fig. S5e–h, *NUPR1* KD remarkably augmented the ratio of yellow to red puncta; if fusion indeed occurred, lysosomes may exhibit an abnormal pH environment or activities.

### *NUPR1* KD inhibited the functions of lysosomes in OSCC cells

To investigate whether *NUPR1* KD inhibited lysosomal functions, the expression levels of lysosome markers located on the surface of the lysosomal membrane, LAMP1 and LAMP2, were examined. The results indicated that *NUPR1* KD inhibited the expression of LAMP1 but not that of LAMP2 in Cal27 and HN6 cells (Fig. 2e, f). The fluorescence intensity of DQ™ Red BSA is positively associated with proteolytic ability based on the proteolysis of BSA conjugates, leading to released protein dequenching.^25^ Lysosomal proteolytic activity was apparently suppressed in *NUPR1*-depleted OSCC cells (Fig. 2g). LysoSensor Green DND-189 dye is an eosinophilic probe that aggregates in acidic organelles, which contain lysosomes, and serves as a pH indicator with an increase in fluorescence intensity dependent on acidification.^25^ *NUPR1* KD decreased the fluorescence intensity of LysoSensor Green DND-189 in Cal27 and HN6 cells (Fig. 2h). Together, these results demonstrated that impairment of autophagic flux by *NUPR1* KD may be mediated by the suppression of lysosomal functions in OSCC cells.

### TFE3 is responsible for NUPR1-mediated autolysosomal processes in OSCC cells

**Fig. 3:**
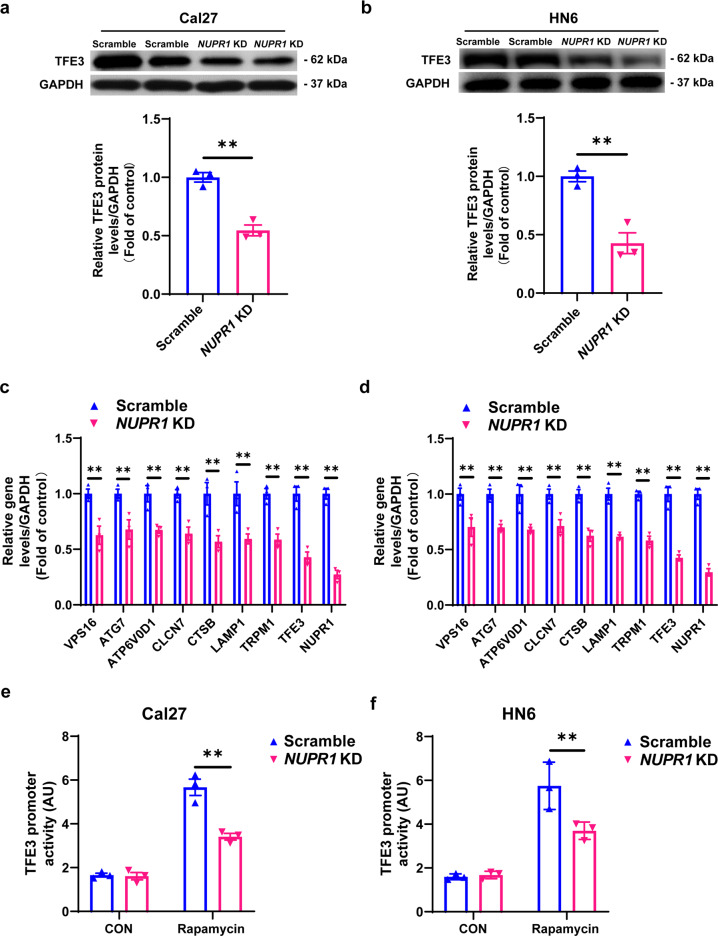
TFE3 was required for NUPR1-mediated autolysosomal processes in OSCC cells. **a**, **b** Immunoblotting analysis of TFE3 in *NUPR1* KD or scrambled Cal27 and HN6 cells; *n* = 3. **c**, **d** The results of *TFE3*-responsive genes involved in autophagic flux were detected by real-time PCR in *NUPR1* KD or scrambled Cal27 and HN6 cells; *n* = 3. **e**, **f** TFE3 transcription activities involved in autophagic flux were detected by Secrete-Pair luminescence assay in *NUPR1* KD or scrambled Cal27 or HN6 cells; *n* = 3; **P < 0.01 vs. the scrambled group

To elucidate the underlying mechanism of NUPR1-mediated autophagy-lysosomal processing, we next analysed the DEPs between Cal27 cells with *NUPR1* KD and scrambled Cal27 cells through proteomic analysis. Interestingly, *NUPR1* KD markedly decreased the expression level of TFE3, as shown by the differentially expressed proteins (Supplementary Table S3). Indeed, TFE3 was markedly downregulated in *NUPR1*-depleted OSCC cells (Fig. 3a, b). More importantly, we confirmed the expression changes in “*TFE3*-responsive genes” involved in autophagic flux by real-time PCR (Fig. 3c, d), suggesting that NUPR1 plays a crucial role in autolysosomal events and may be related to TFE3. We performed luciferase reporter assays to detect the relationship between NUPR1 and TFE3. Recently, NUPR1 was reported as an important transcription factor that regulates gene transcription.^15^ As shown in Fig. 3e, f, TFE3 promoter activity was distinctly increased in OSCC cells treated with the autophagy agonist rapamycin (0.1 µM) for 24 h but was substantially inhibited in *NUPR1*-depleted OSCC cells compared to control cells. Together, these results demonstrated that NUPR1 maintains autophagic flux by increasing TFE3 promoter activity and activating TFE3 transcription during OSCC progression. Moreover, correlation and simple linear regression of NUPR1 expressions with TFE3 expressions in 88 samples were analysed and calculated, and a positive correlation between NUPR1 and TFE3 was identified (Supplementary Fig. S6).

**Fig. 4:**
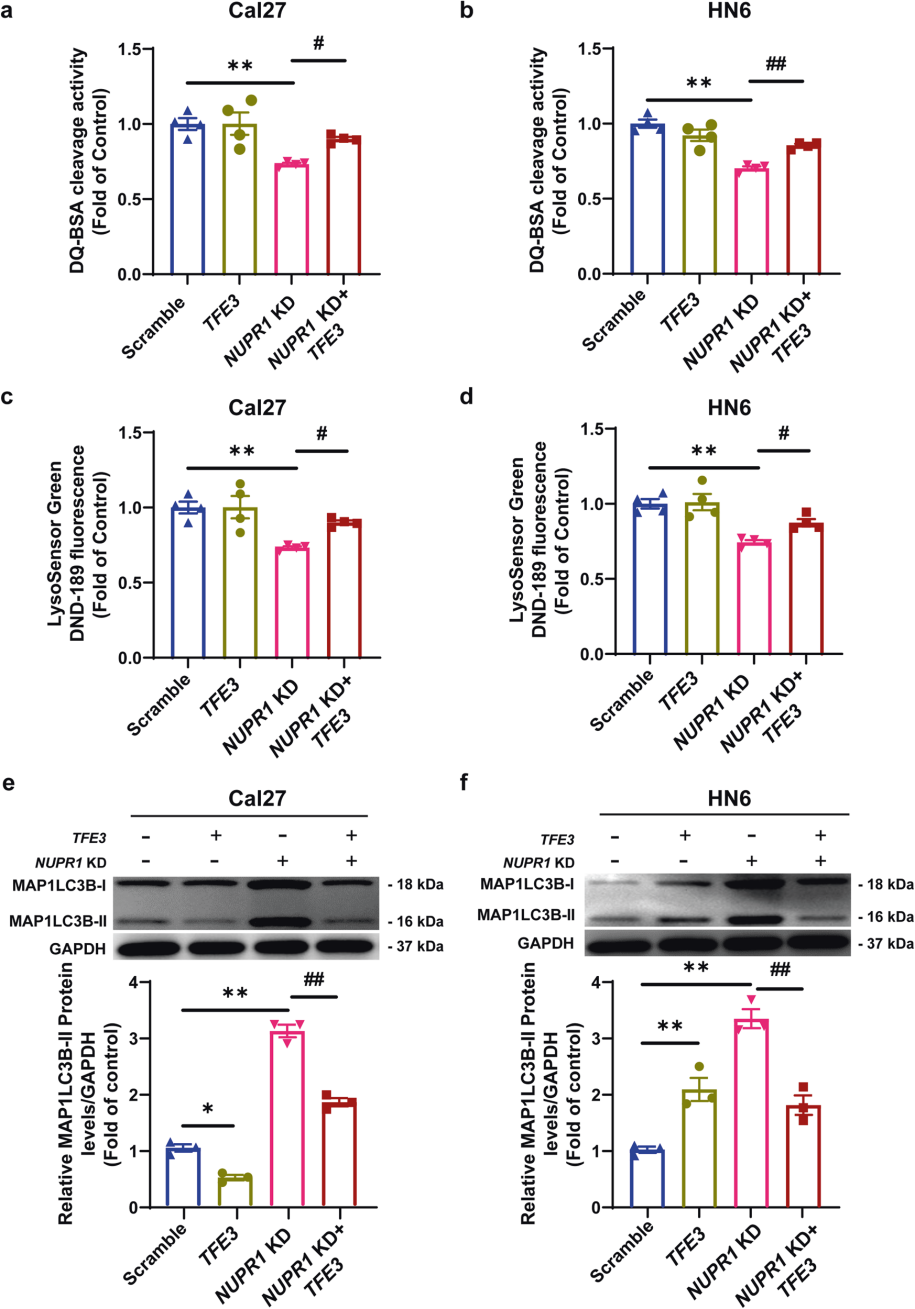
*TFE3* overexpression rescued the *NUPR1* KD-inhibited autophagic flux in OSCC cells. **a**, **b** DQ-BSA or **c**, **d** LysoSensor DND-189 fluorescence intensity was detected in *NUPR1* KD or scrambled Cal27 and HN6 cells transfected with *TFE3* plasmid or a control plasmid for 24 h; *n* = 4. **e**, **f** Immunoblotting analysis of MAP1LC3B and SQSTM1 in *NUPR1* KD or scrambled Cal27 and HN6 cells transfected with *TFE3* plasmid or a control plasmid for 24 h; *n* = 3; *P < 0.05, **P < 0.01 vs. the scrambled group. ^#^*P* < 0.05, ^##^*P* < 0.01 vs. *NUPR1* KD group

We further overexpressed *TFE3* in OSCC cells with or without *NUPR1* KD. DQ™ Red BSA and LysoSensor Green DND-189 dye detection assays showed that overexpression of *TFE3* could significantly rescue the damage to lysosome function caused by *NUPR1* KD in Cal27 and HN6 cells (Fig. 4a–d). Moreover, MAP1LC3B-II and SQSTM1 accumulation was also markedly decreased with *TFE3* overexpression in *NUPR1*-depleted OSCC cells (Fig. 4e, f).

### NUPR1 promoted OSCC progression by activating TFE3-dependent autophagy in vitro and in vivo

**Fig. 5:**
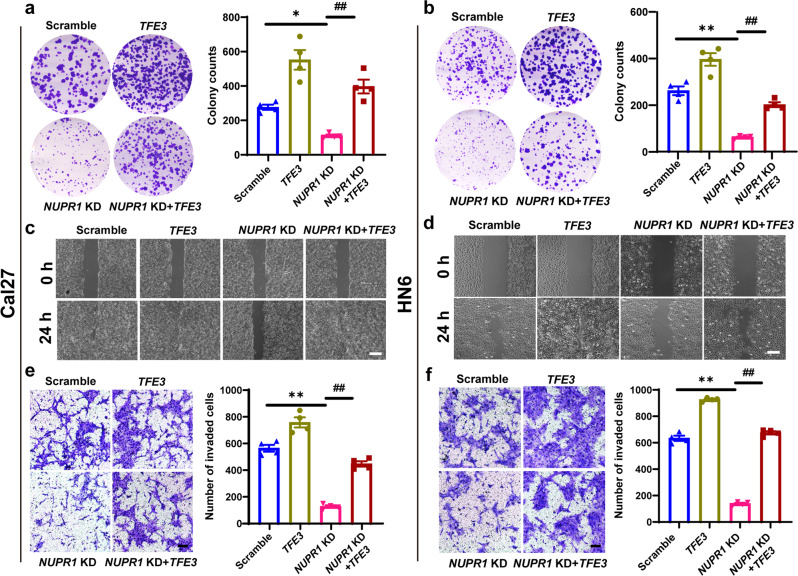
*TFE3* overexpression antagonised the *NUPR1* KD-induced inhibition of OSCC cell proliferation and metastasis. **a**, **b** The colony formation results for *NUPR1* KD or scrambled Cal27 and HN6 cells transfected with *TFE3* plasmid or a control plasmid; *n* = 4. **c**, **d** The migration results for of *NUPR1* KD or scrambled Cal27 and HN6 cells transfected with *TFE3* plasmid or a control plasmid; Scale bar: 100 μm. **e**, **f** The invasion results for *NUPR1* KD or scrambled Cal27 and HN6 cells transfected with *TFE3* plasmid or a control plasmid. Scale bar = 80 μm; magnification; *n* = 4; 40×; *P < 0.05, **P < 0.01 vs. the scrambled group, ^##^*P* < 0.01 vs. the *NUPR1* KD group

**Fig. 6:**
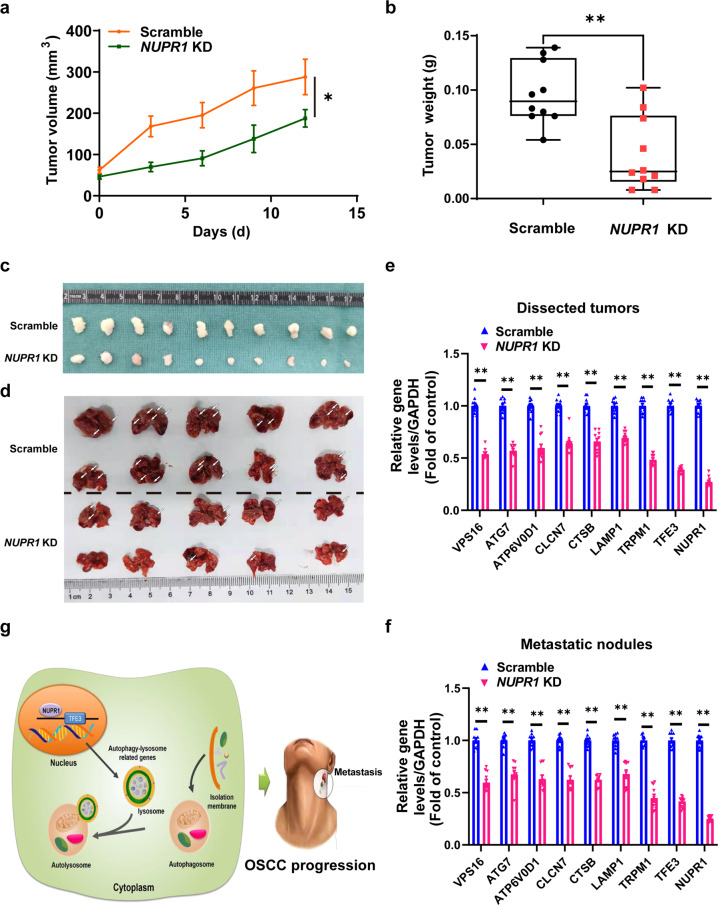
*NUPR1* KD inhibited aggressiveness of OSCC in nude mice. **a**, **b** Calculated volume and weight of xenograft tumours generated by *NUPR1* KD or scrambled Cal27 cells subcutaneously inoculated into mice. *n* = 10. **c** Dissected tumours were photographed. *n* = 10. **d** Typical lung tissues with observable metastatic nodules from nude mice after tail vein injection of *NUPR1* KD or scrambled Cal27 cells. *n* = 10. **e**, **f** The expression of *TFE3*-responsive genes involved in autophagic flux was detected by real-time PCR in dissected tumours or metastatic nodules of lung tissues; *n* = 10. *P < 0.05, **P < 0.01 versus the scrambled group. **g** Schematic diagram illustrating the underlaying molecular mechanisms of NUPR1/TFE3 axis-mediated autophagic flux in OSCC progression

We then performed *TFE3* overexpression to observe the change in the progression abilities of *NUPR1*-deficient OSCC cells in vitro. Plate colony formation assays and wound healing and Matrigel-coated Transwell assays demonstrated that *TFE3* overexpression partially reversed the inhibitory effects of *NUPR1* KD-mediated OSCC cell migration and invasion (Fig. 5a–f). Importantly, we also found that *NUPR1* KD resulted in an obvious reduction in the volume and weight of tumours formed by Cal27 cells inoculated in the subcutis of nude mice (Fig. 6a–c). Moreover, *NUPR1* KD also inhibited the formation and growth of the metastatic nodules(Fig. 6d). Additionally, *NUPR1* KD efficiently suppressed *TFE3* and “*TFE3-*responsive genes” expression in the xenograft tumour models (Fig. 6e). The same results were obtained in the assessment of metastatic nodules (Fig. 6f). Overall, these findings indicated that the inhibition of OSCC progression caused by *NUPR1* KD is dependent on the suppression of TFE3 activity.

## Discussion

The high metastasis and recurrence rates, which are associated with a high mortality rate, are some of the main features of OSCC noted recently; due to a lack of powerful treatment strategies, this cancer presents a significant burden to human health.^3,4^ Seeking the key molecules that regulate the recurrence and metastasis of OSCC is an important direction and prerequisite for developing effective therapeutic drugs. In this study, we first demonstrated (i) NUPR1 was identified as a pivotal protein that was remarkably augmented in LNM patients and positively related to OSCC metastasis and poor prognosis with the high-sensitivity quantitative label-free quantitative proteomics analysis. (ii) Lysosomal dysfunction but not other key steps disruption (autophagosome formation or maturation, and autophagosome-lysosome fusion) is a crucial cause of NUPR1 *KD*-induced autophagic flux impairment and inhibition of OSCC cell proliferation and metastasis. (iii) NUPR1 maintained autophagic flux by increasing TFE3 promoter activities and activating TFE3 transcription in OSCC progression. These findings, therefore, broaden new insights regarding a potential therapeutic target of NUPR1-TFE3-dependent autophagy in OSCC progression.

Most tumour tissue specimens archived in hospitals for pathologic diagnosis are FFPE specimens, which represent the pinpoints of tumour tissue and generally contain rich clinical data. Proteomics is an efficient tool to investigate discrepancies in protein expression in different tumour tissues, especially in FFPE tissues, and to further analyse the pathogenesis of diseases.^26^ In this study, FFPE tumour samples of OSCC patients with or without LNM were comparatively profiled with high-sensitivity quantitative proteomics analysis. NUPR1 was identified and verified to have potential prognostic value; it was highly expressed in OSCC patients with LNM and was significantly associated with low pathological differentiation grades, regional LNM, clinical stage, and decreased overall survival time.

NUPR1 is a transcription factor that is dependent on a basic helix-loop-helix structure present at its C-terminus, and its function may be mediated by various kinases, factors or cellular stressors.^15^ NUPR1 was reported to result in cancer development and progression over twenty years ago and has been found to be involved in multiple aspects of cancer, especially transcription regulation of cancer cells.^15^ NUPR1 was found to promote metastasis and chemotherapeutic resistance in some cancers, such as breast, lung, and colorectal cancer.^27^ Recently, Jiang *et al*. reported that upregulating the activation of NUPR1 can significantly promote epithelial-mesenchymal transition (EMT) of SCC-9 and Tca8113 OSCC cells.^28^ Huang et al. also found that enhancing the expression level of NUPR1 promoted the growth and invasiveness of SCC-9 and HSC-2 OSCC cells.^29^ These findings indicated that NUPR1 serves as a key factor mediating OSCC development and progression, which is in accordance with our results and further supports the possibility of targeting NUPR1 for OSCC treatment.

Autophagy plays a crucial role in the maintenance of intracellular homoeostasis in response to cellular stress.^16^ Autophagy is generally considered a mechanism that allows cancer cells to survive under conditions of stress such as hypoxia and starvation.^16^ The transcription factor NUPR1 serves as a master regulator of cellular clearance through promoting autophagy process.^15^ Recently, Wu *et al*. found that *NUPR1* knockdown blocked autophagic flux and increased apoptosis of liver cancer cell,^30^ and Wang *et al*. reported that *NUPR1* depletion induced premature senescence in breast cancer cells by impairing the autolysosomal process.^31^ Moreover, NUPR1 maintains autophagic flux and is required for the development of lung cancer.^16^ In accordance with these results, in this study, we revealed that several proteins were participated in autophagic flux in *NUPR1* KD cells via proteomic and bioinformatic analysis. Autophagic flux is considered to be a multistage cascade that generally includes the phagophore form phase, the expansion of phagophores into an autophagosome stage, the fusion of autophagosomes with lysosomes and finally degradation in lysosomes.^25^ Importantly, we further found that *NUPR1* KD substantially disrupted lysosomal function but did not disturb other key steps of autophagic flux. Thus, lysosomal dysfunction is a crucial cause of *NUPR1* KD-induced autophagic flux blockage and inhibition of OSCC cell proliferation and metastasis.

TFE3 has been recognised as a key regulator of the genes expression that are correlated with autophagosome and autolysosome formation and degradation.^11,20^ Emerging evidence indicates that TFE3-dependent autophagy–lysosome activation is required for lysosomal function and maintains pH, which significantly promote the progression of cancers such as renal cancer, pancreatic cancer, and epithelioid haemangioendothelioma.^18,32^ Interestingly, our data indicated that TFE3 expression was markedly decreased following *NUPR1* KD and that *TFE3* overexpression antagonised the inhibition of lysosome function caused by *NUPR1* KD in OSCC cells. NUPR1 can directly regulate genes transcription by combining with platelet-derived growth factor subunit A (PDGFA), synaptosomal-associated protein 25 (SNAP25), and other gene promoters.^27^ In our study, we found that NUPR1 could substantially enhance TFE3 promoter activity, and we also found a positive correlation between NUPR1 and TFE3 in OSCC tissues. This study is the first to examine these aspects of OSCC progression, and the findings indicate the existence of a NUPR1/TFE3 link.

In summary, by combining high-sensitivity label-free quantitative proteomics and TMT-based quantitative proteomics, we speculate that the NUPR1-TFE3 axis-mediated autolysosomal clearance pathway may contribute to OSCC development and that this pathway could be exploited for the prevention and therapy of OSCC (Fig. 6g).

## Materials and methods

### Ethics statement

OSCC and oral mucosa tissue samples were obtained from the Department of Oral and Maxillofacial Surgery, the Second Xiangya Hospital of Central South University. All participants provided written informed consent before the study. The protocols were approved by the Clinical Research Ethics Committee of the Second Xiangya Hospital of Central South University (NO. JBWKQA001), and the animal experiments were approved by the ethics statement of the Shanghai Jiao Tong University Institute Animal Care and Use Committee.

### Cell culture and *NUPR1* KD OSCC cell line construction

The OSCC cell lines HN4, HN6, and HN30 were kindly gifted by Professor Mao Li from the University of Maryland. SCC9, SCC25 and Cal27 cell lines were purchased from the American Type Culture Collection (ATCC, USA). All these cells except SCC9 and SCC25 were cultured in DMEM (Thermo Fisher, 11995040) supplemented with 10% FBS (Thermo Fisher, 10091155) and 1% (v:v) penicillin/streptomycin (Sigma, P4333) at 37 °C in a humidified 5% CO_2_ atmosphere. While SCC9 and SCC25 cells were kept in DMEM/F12 medium. To investigate the role of NUPR1 in OSCC development, stable *NUPR1* KD HN6 and Cal27 cell lines were constructed at Shanghai GeneChem Co., Ltd.

### FFPE tissue preparation and high-sensitivity label-free quantitative proteomics analysis, TMT quantitative proteomic analysis

Detailed information of quantitative proteomics analysis is shown in the Supplementary Method.

### Tissue microarray (TMA), Cell migration, invasion assay and colony formation assay

For the methods to determine the OSCC progression in vitro are shown in the Supplementary Method.

### Western blot analysis, Plasmid or RFP-GFP-LC3B lentivirus transfection, Immunofluorescence analysis, DQ-BSA proteolytic activity assay, LysoSensor Green DND-189 staining, Real-Time PCR analysis, and Secrete-Pair luminescence assay

The protocols are shown in the Supplementary Method.

### Xenograft mouse experiments and in vivo metastasis assay

The tumour xenograft model was established with 1 × 10^6^ Cal27 cells according to our recent report.^18^ The BALB/c nude mice were divided into 2 groups (*n* = 10 per group): (a) scrambled Cal27 cells and (b) stable *NUPR1* KD Cal27 cells. Tumour volumes and tumour weight measurements were performed as previously reported.^11^ For the experimental metastasis assay, 1 × 10^6^ cells (scrambled Cal27 cells or *NUPR1* KD Cal27 cells) in 100 μl normal saline were injected into the caudal vein of nude mice (*n* = 10 per group). The injected mice were euthanized after 7 weeks as previously reported.^33^ The lungs were removed, and subsequent experiments were performed. The mRNA levels of *NUPR1* and *TFE3*-responsive genes in tumours and metastatic nodules were quantified.

### Statistical analysis

Data were analysed using the student’s *t* test or non-parametric Mann–Whitney *U* Test and one-way analysis of variance (ANOVA) in GraphPad Prism version 8. Mann–Whitney *U* tests were used to analyse the associations between TMA scores and clinical paraments as well as the puncta immunofluorescence study. The log-rank test was used to assess the survival differences and Kaplan–Meier survival analyses were used to estimate the prognostic and diagnostic value. The correlation between NUPR1 and TFE3 was determined by Pearson analysis. *P* < 0.05 was considered statistically significant. All values are expressed as the mean ± SE.

## Supplementary information

SUPPLEMENTAL MATERIAL

## Data Availability

All data are available from the corresponding author upon reasonable request.
