# Correction to: NUPR1 promotes the proliferation and metastasis of oral squamous cell carcinoma cells by activating TFE3-dependent autophagy

**DOI:** 10.1038/s41392-022-01154-0

**Published:** 2022-09-16

**Authors:** Tengfei Fan, Xiaoning Wang, Sheng Zhang, Ping Deng, Yi Jiang, Yidan Liang, Sheng Jie, Qing Wang, Chuwen Li, Guocai Tian, Zhen Zhang, Zhenhu Ren, Bo Li, Yanrong Chen, Zhijing He, Yan Luo, Mingliang Chen, Hanjiang Wu, Zhengping Yu, Huifeng Pi, Zhou Zhou, Zhiyuan Zhang

**Affiliations:** 1 Department of Oral and Maxillofacial-Head Neck Oncology, Shanghai Ninth People’s Hospital, Shanghai Jiao Tong University School of Medicine; College of Stomatology, Shanghai Jiao Tong University; National Center for Stomatology; National Clinical Research Center for Oral Diseases; Shanghai Key Laboratory of Stomatology; Research Unit of Oral and Maxillofacial Regenerative Medicine, Chinese Academy of Medical Sciences, Shanghai, China; 2 Department of Oral and Maxillofacial Surgery, Zhang Zhiyuan Academician Workstation, Hainan Western Central Hospital, Shanghai Ninth People’s Hospital, Danzhou, Hainan China; 3 Department of Oral and Maxillofacial Surgery, The Second Xiangya Hospital of Central South University, Changsha, Hunan China; 4 Department of Oral Pathology, Shanghai Ninth People’s Hospital, Shanghai Jiao Tong University School of Medicine, Shanghai, China; 5 Department of Occupational Health, Third Military Medical University, Chongqing, China; 6 Department of Pathology, The Second Xiangya Hospital of Central South University, Changsha, Hunan China; 7 School of Medicine, Guangxi University, Nanning, Guangxi Zhuang Autonomous Region China; 8 Department of Oral and Maxillofacial Surgery, Affiliated Hospital of Guilin Medical University, Guilin, Guangxi Zhuang Autonomous Region China; 9 Institute of Pathology and Southwest Cancer Centre, Southwest Hospital, Third Military Medical University, Chongqing, China; 10 Department of Emergency Medicine, First Affiliated Hospital and Department of Environmental Medicine, School of Public Health, School of Medicine, Zhejiang University, Hangzhou, Zhejiang China

**Keywords:** Head and neck cancer, Head and neck cancer

Correction to: *Signal Transduction and Targeted Therapy* 10.1038/s41392-022-00939-7, published online 25 April 2022

In the process of checking the raw data^1^, the authors noticed several inadvertent mistakes occurring in Fig. 5a, b, c, d, f that need to be corrected after online publication of the article. During the preparation of Fig. 5, the representative image showing *TFE3* overexpression antagonized the *NUPR1* KD-induced inhibition of OSCC cell proliferation and metastasis, were pasted and placed by mistake. The correct results should be as shown below. The authors apologize for these oversights and declare that these corrections do not affect the description, interpretation, or conclusions detailed in the original manuscript.

**Fig. 5:**
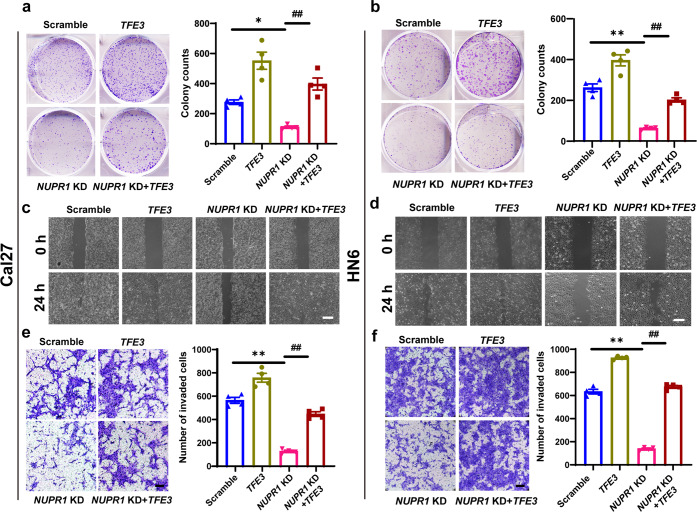
**a**, **b** Representative images of colony formation and quantitative analysis results in *NUPR1* KD or scrambled Cal27 and HN6 cells transfected with *TFE3* plasmid or a control plasmid for 24 h; *n* = 4. **c**, **d** The migration results for of *NUPR1* KD or scrambled Cal27 and HN6 cells transfected with *TFE3* plasmid or a control plasmid; scale bar: 100 μm. **e**, **f** The invasion results for *NUPR1* KD or scrambled Cal27 and HN6 cells transfected with *TFE3* plasmid or a control plasmid. Scale bar = 80 μm; magnification; *n* = 4; 40**×**;
