# Supplementary material for: NUPR1 promotes the proliferation and metastasis of oral squamous cell carcinoma cells by activating TFE3-dependent autophagy
Source: Signal Transduct Target Ther. 2022 Apr 25;7:130. doi: 10.1038/s41392-022-00939-7 (PMC9035452; doi:10.1038/s41392-022-00939-7)
Supplement: Supplementary file 1 — SUPPLEMENTAL MATERIAL [file 41392_2022_939_MOESM1_ESM.pdf]

Supplementary Materials for

NUPR1 promotes the proliferation and metastasis of oral squamous cell carcinoma cells by activating TFE3-dependent autophagy

Tengfei Fan, Xiaoning Wang, Sheng Zhang, Ping Deng, Yi Jiang, Yidan Liang, Sheng Jie, Qing Wang, Chuwen Li, Guocai Tian, Zhen Zhang, Zhenhu Ren, Bo Li, Yanrong Chen, Zhijing He , Yan Luo, Mingliang Cheng, Hanjiang Wu, Zhengping Yu, Huifeng Pi, Zhou Zhou, Zhiyuan Zhang

Correspondence to: Huifeng Pi, E-mail: pihuifeng2010@163.com; Zhou Zhou, E-mail: lunazhou@zju.edu.cn; Zhiyuan Zhang, E-mail: zhzhy0502@163.com

This PDF file includes:

Materials and Methods

Figures. S1 to S6

Tables S1 to S6

Materials and Methods

Tissue microarray (TMA)

For all included patients, TMA was taken. TMA analysis and IHC staining were performed by Wuhan Servicebio Technology Co., Ltd. Tissue sections were incubated with NUPR1 antibody (Abcam, ab234696). Two expert pathologists examined and scored the TMA independently without any sample information.

FFPE tissue preparation and high-sensitivity label-free quantitative proteomics analysis

FFPE tumour tissue preparation and label-free were performed as previously reported.[1] FFPE biobank specimens (5 serial sections, 10 μM thick) were deparaffinized by xylene (50 °C, 5 min) and washed in 1 ml absolute ethanol twice. Areas containing 70 % or more tumor were dissected from the slide according to a same tumor slide as reference which was performed by hematoxylin stained. Lysis was extracted in 4 % SDS at 99 °C for 60 min and accompanied by 15 min sonication. Proteins in the cleared lysate were reduced with 10mM DTT for 30 min and alkylated with 55 mM iodoacetamide for an additional 30 min. 100 μg proteins were purified by acetone precipitation and the protein pellets resolved in 100 mL 6 M urea/2 M thiourea. Protein lysis was digested with LysC buffer for 3 h and 4 volumes of 50 mM ammonium bicarbonate buffer, 1 mg trypsin were added for tryptic digestion overnight. The next day, digestion was stopped by 1 % TFA. Peptides were finally desalted on C18 StageTips, suspended in 10 mL 2 % acetonitrile, 0.1 % TFA and kept at -20 °C until LC-MS/MS analysis. Samples from the two patient groups were measured in random order. Peptide samples were diluted to 1 μg/μl on-board buffer, the sample volume was set to 5 μl, and the scanning mode was 120 min. Scan the peptides with a mass-to-charge ratio of 350-1500 in the sample. Prepare mobile phase A solution (98 % water, 2 % ACN, 0.1 % FA), B solution (98 % ACN, 2 % water, 0.1 % FA), pre-column (300 μm×0.5 mm, 3 μm), analytical column (3 μm, 75 μm×150 mm, Welch Materials, Inc), spray voltage 1.9 KV, peptides separated by liquid phase are ionized by nanoESI source and enter into tandem mass spectrometer Q-Exactive HFX (Thermo Fisher Scientific, San Jose , CA) detection. MS raw files were processed with the MaxQuant software. The integrated Andromeda search enginewas used for peptide and protein identification at an FDR of less than 1 %. The human UniProtKB database was used as forward database and the automatically generated reverse database for the decoy search. Label free protein quantification was performed using the

MaxLFQ algorithm. The mass spectrometry proteomics data of FFPE tissues have been deposited to the ProteomeXchange Consortium (http://proteomecentral. proteomexchange.org) via the iProX partner repository with the dataset identifier PXD030554.

Colony formation assay

The colony formation assay was performed as described in a previous report.[2] Briefly, 1 ml 0.8% agar solution in medium was added and solidified in six-well plates and then overlaid with 1 ml 0.3% agar solution in medium containing 2000 cells. After 10 days, the cell colonies were stained with 0.5 ml iodonitrotetrazolium violet (INT, 0.5 mg/ml; Merck, I7375-1G). The cell colonies were then counted and photographed.

Cell migration assay

After the cells were seeded in 6-well plates to 100 % confluency, a wound was induced by scratching the cell cultures with a 5 μl pipette tip. Following three rinses with phosphate-buffered saline (PBS) to remove detached cells, adherent cells were cultured in a medium without serum. Four random fields of each well were imaged immediately after injury and 24 h later using a microscope (Nikon Corporation, Tokyo, Japan) at ×10 magnification. The distance between the wound edges was calculated using Nikon Application Suite software.

Cell invasion assay

Cell invasion assays were performed using Transwell chambers (Corning, 3422) as described in a previous report.[3] A total of 2×10^4 cells in serum-free medium were inoculated into Matrigel-coated upper chamber, and 600 μl medium with 10% FBS was inoculated into the lower chamber. After crystal violet staining, the cells that crossed the membrane were counted and photographed.

TMT quantitative proteomic analysis

Stable *NUPR1* KD Cal27 cells and scrambled control were dissociated in SDT buffer (4 % SDS， 100 mM Tris-HCl， 1mM DTT， pH 7.6). The proteomics analysis, including protein digestion, TMT labelling, fractionation, LC-MS/MS analysis, protein identification, and protein quantitation was performed by Applied Protein Technology (Shanghai, China) as described in our previous study.[4]

Western blot analysis

The cells were dissociated in RIPA buffer (Beyotime, P0013B). The sample protein was electrophoresed by SDS-PAGE and transferred onto PVDF membranes. Membranes were subsequently probed with primary antibody at 4 °C overnight. Then, the membranes were incubated with the secondary antibody for 1 h at room temperature, and the bands acquired from membranes were scanned with a ChemiDoc XRS^+ System. The signals of the bands were analysed with Image Lab (Bio-Rad). All the antibodies used are listed in Table S4.

Plasmid transfection

Plasmid transfection assays were performed as previously reported with Lipofectamine™ 3000 Reagent (Thermo Fisher, L3000015).[5] The cells were transfected with 2 μg/mL BacMam LC3B-GFP (Thermo Fisher, P36235), ATG5-shRNA plasmid (GenePharma, China) or TFE3 plasmid (GenePharma, China) in parallel with a pcDNA 3.0 plasmid (GenePharma, China) as a control group. After transfection for 24 h, the cells were used for subsequent experiments.

Immunofluorescence analysis

Immunofluorescence analysis was performed according to our previous report.[6] The cells were fixed with 4% paraformaldehyde (Beyotime, P0099) and infiltrated with 0.25% Triton X-100 (Sangon Biotech, A110694-0100). The cells were blocked with 10% BSA in PBS and incubated with primary antibody at 4 °C overnight, followed by fluorescent secondary antibodies based on the source of the primary antibody. The stained slides were examined using a confocal laser scanning microscope (Leica TCS SP8, Germany) equipped with a 63 × or 40 × oil objective. The colocalization coefficient was calculated using ImageJ software. At least 30 cells were counted for each experiment. All the antibodies used are listed in Table S5.

RFP-GFP-LC3B assay

RFP-GFP-LC3B assays were performed with the PremoTM Autophagy Tandem Sensor RFP-GFP-LC3B kit (Invitrogen, P36239) after lentivirus transfection as previously reported.[7] Briefly, RFP-GFP-LC3B lentivirus was added to glass-bottom dishes inoculated with 2×10^5 Cal27 or HN6 cells. After 24 h, subsequent experiments were performed. All samples were examined under a confocal laser scanning microscope (Leica TCS SP8, Germany).

DQ-BSA proteolytic activity assay

A total of 1 × 10^4 cells were cultured in 96-well plates for lysosomal proteolysis activity detection. Cells were incubated with 10 μg/mL DQ™ Red BSA (Invitrogen, D-12051) at 37 °C for 6 h. The fluorescence intensity of the cells was detected by an Infinite™ M200 Microplate Reader (excitation: 590 nm, emission: 620 nm).[7]

LysoSensor Green DND-189 staining

To detect the lysosomal pH, the cells were incubated in medium with 1 μM LysoSensor Green DND-189 (Invitrogen, L7535) for 5 min at 37 °C. The fluorescence intensity of cells was detected by an Infinite™ M200 Microplate Reader (excitation: 485 nm, emission: 530 nm).[7]

Real-Time PCR analysis

Total RNA was isolated with RNAiso Plus (TaKaRa, 9109) as previously described.[8] First-strand cDNA was synthesized from total RNA using SuperScript IV Reverse Transcriptases (Thermo Fisher, 18090050). Real-time PCR was detected by SYBR Green PCR Master Mix (Thermo Fisher, 4309155) using the LightCycler 96 System (Roche). The qPCR primers are listed in Table S6.

Secrete-Pair luminescence assay

Dual-reporter promoter clones or controls were transfected into Cal27 and HN6 cell lines. The indicated scrambled and stable NUPR1 KD cells were transfected with the pEZX-PG04-TFE3 promoter Gaussia luciferase/secreted alkaline phosphatase (GeneCopoeia, HPRM41027-PG04). After 24 h, these cells were treated with rapamycin (0.1 μM) for another 24 h. The Secrete-Pair Dual Luminescence Assay kit (GeneCopoeia, SPDA-D010) was used to detect TFE3 promoter luciferase activity as previously reported.[9]

BALB/c nude mice

BALB/c nude mice (male, 4 weeks and approximately 20 g) were purchased from the Shanghai Laboratory Animal Center (Shanghai, China) and bred in specific-pathogen-free (SPF) facilities at Shanghai Ninth People's Hospital.

REFERENCE

1 Buczak, K. et al. Spatially resolved analysis of FFPE tissue proteomes by quantitative mass spectrometry. *Nat. Protoc.* **15**, 2956-2979 (2020).

2 Lin, C. et al. Nerve growth factor (NGF)-TrkA axis in head and neck squamous cell carcinoma triggers EMT and confers resistance to the EGFR inhibitor erlotinib. *Cancer. Lett.* **472**, 81-96 (2020).

3 Liang, Y. et al. Cadmium promotes breast cancer cell proliferation, migration and invasion by inhibiting ACSS2/ATG5-mediated autophagy. *Environ. Pollut.* **273**, 116504 (2021).
4 Xi, Y. et al. Inhibition of SERPINA3N-dependent neuroinflammation is essential for melatonin to ameliorate trimethyltin chloride-induced neurotoxicity. *J. Pineal. Res.* **67**, e12596 (2019).
5 Deng, P. et al. Bisphenol A promotes breast cancer cell proliferation by driving miR-381-3p-PTTG1-dependent cell cycle progression. *Chemosphere.* **268**, 129221 (2021).
6 Pi, H. et al. SIRT3-SOD2-mROS-dependent autophagy in cadmium-induced hepatotoxicity and salvage by melatonin. *Autophagy.* **11**, 1037-1051 (2015).
7 Pi, H. et al. Enhancing lysosomal biogenesis and autophagic flux by activating the transcription factor EB protects against cadmium-induced neurotoxicity. *Sci. Rep.* **7**, 43466 (2017).
8 Pi, H. et al. Transcription factor E3 protects against cadmium-induced apoptosis by maintaining the lysosomal-mitochondrial axis but not autophagic flux in Neuro-2a cells. *Toxicol. Lett.* **295**, 335-350 (2018).
9 Liu, J. et al. NUPR1 is a critical repressor of ferroptosis. *Nat. Commun.* **12**, 647 (2021).

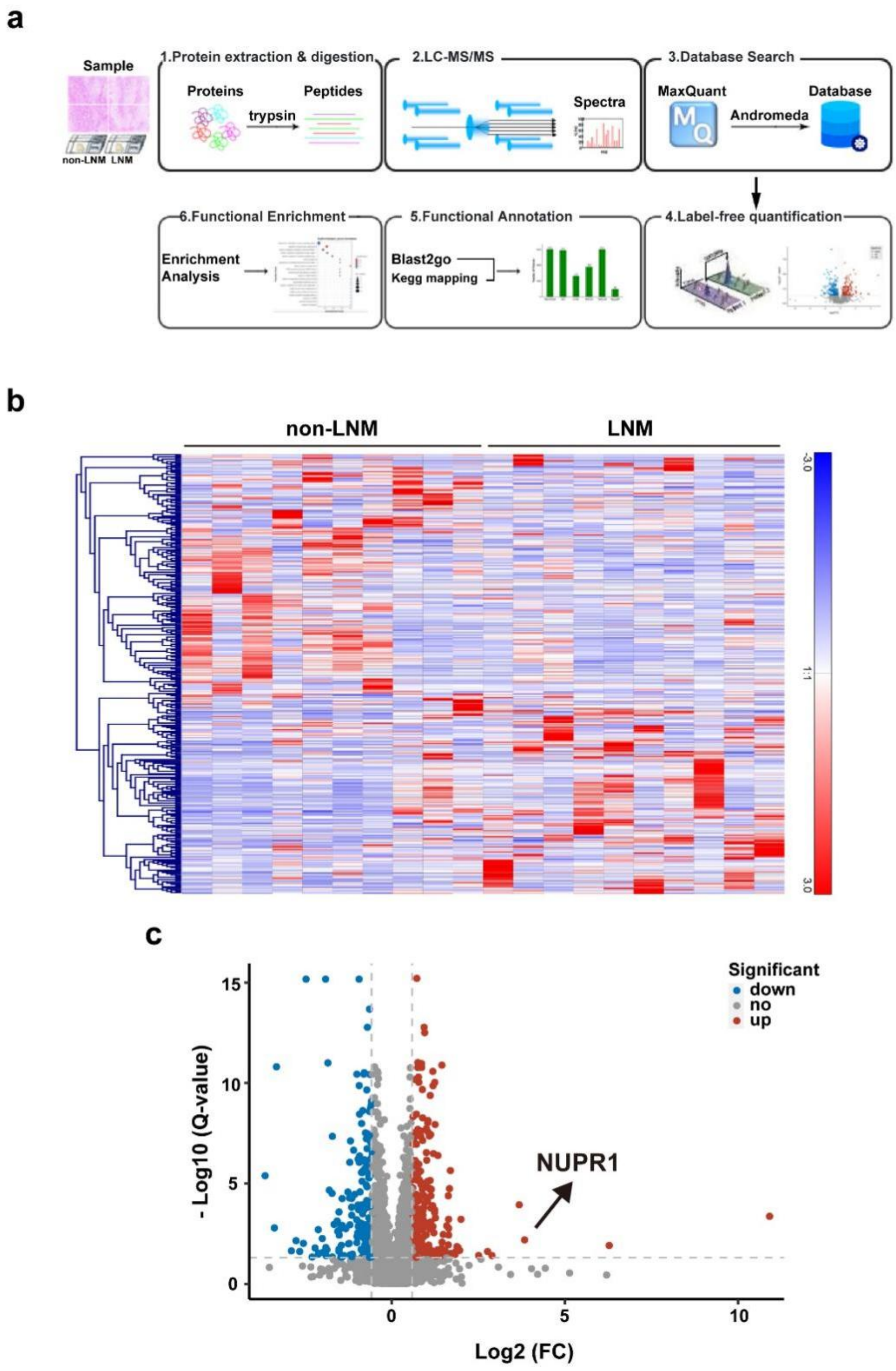

Figure. S1. High-sensitivity label-free quantitative proteomics analysis of FFPE tumour samples derived from non-LNM and LNM OSCC patients. **a** Summary of the shotgun proteomics workflow applied to FFPE tumours from OSCC patients in this study. **b** Clustering analysis of differentially expressed proteins. Blue, low expression; red, high expression; n =10. **c** Volcano plot of proteomics results. Red plots represent significantly upregulated proteins, whereas blue plots represent significantly downregulated proteins in non-LNM patients. Dashed lines indicate the significance threshold (FDR < 0.05, | Log FC | ⩾ 2).

a

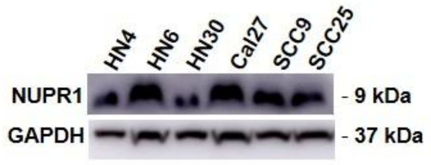

b

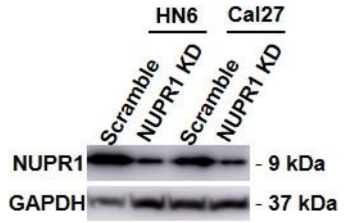

Figure. S2. *NUPR1* KD decreased NUPR1 expression in OSCC cells. **a** Immunoblotting analysis of NUPR1 expression in different OSCC cell lines. **b** Immunoblotting analysis of NUPR1 expression in *NUPR1* KD or scrambled Cal27 and HN6 cells.

a

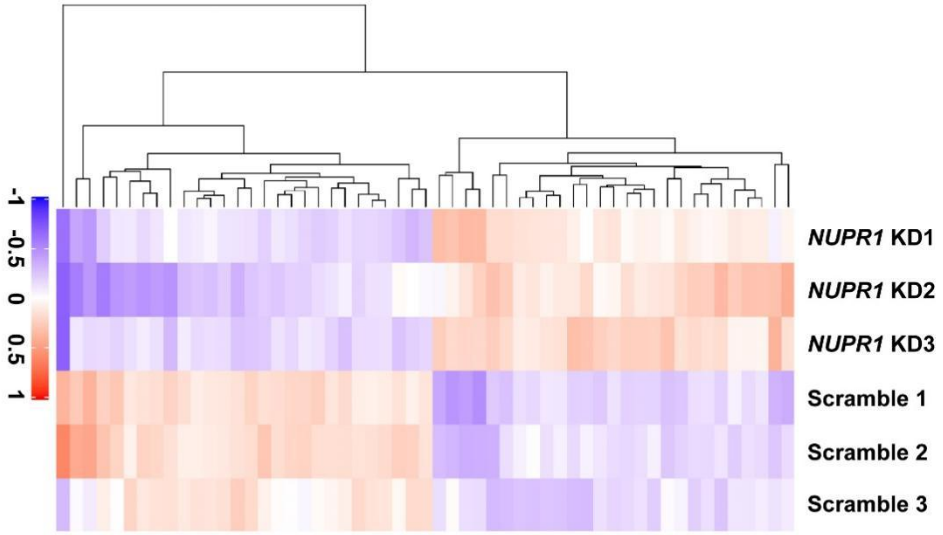

b

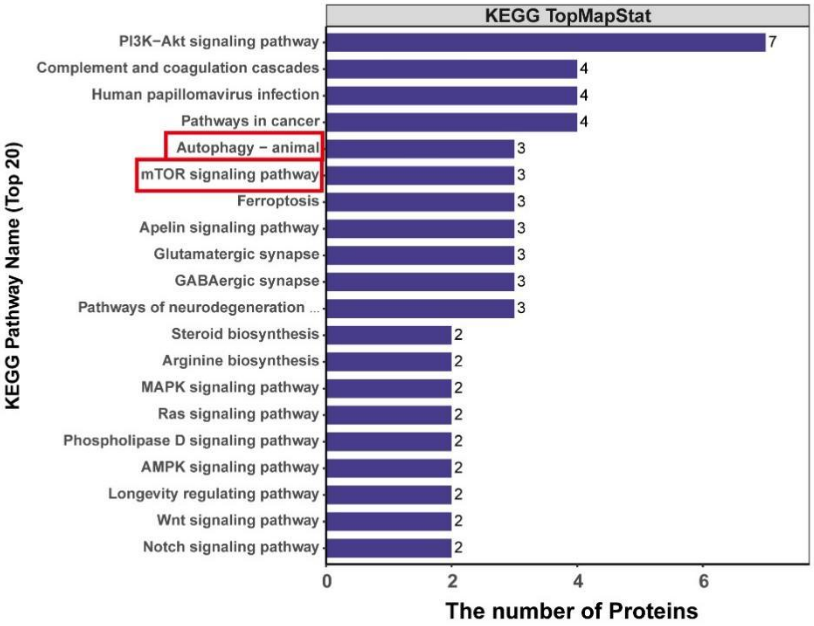

Figure. S3. TMT-based quantitative proteomics analysis indicated the crucial role of autophagy in NUPR1-mediated OSCC progression. **a** Clustering analysis of differentially expressed proteins analysed by TMT-based proteomics in *NUPR1* KD Cal27 cells compared with scrambled control; n=3. **b** KEGG pathway enrichment analysis.

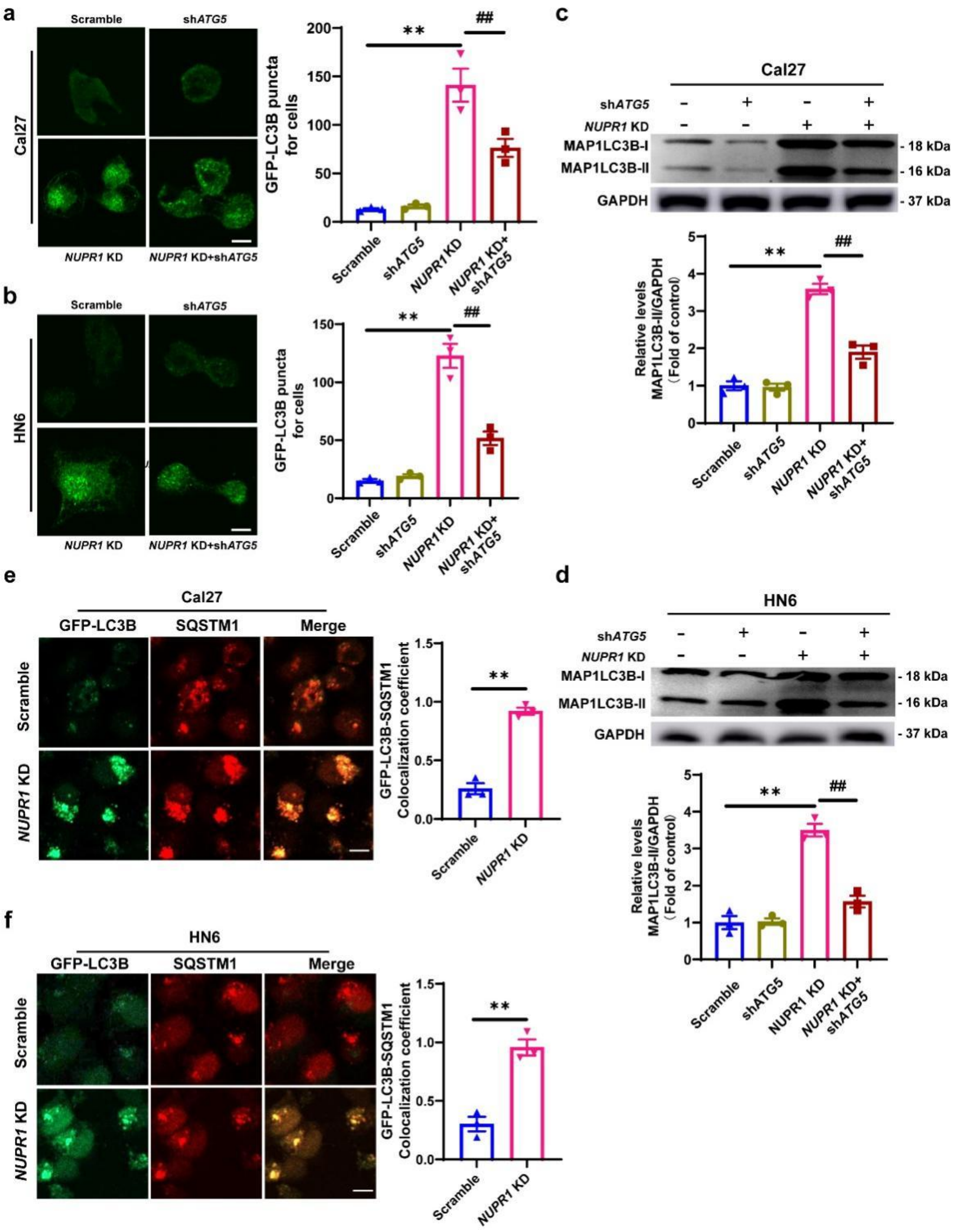

Figure. S4. *NUPR1* KD did not affect autophagy induction or autophagosome maturation in OSCC cells. **a-b** GFP-LC3B puncta formation in *NUPR1* KD or scrambled Cal27 and HN6 cells transfected with or without *ATG5* shRNA; n=3; Scale bar = 10 μm. **c-d** Immunoblotting analysis of MAP1LC3B in *NUPR1* KD or scrambled Cal27 and HN6 cells transfected with or without *ATG5* shRNA; n=3. **e-f** Representative immunofluorescence images and colocalization coefficients of GFP-LC3B and SQSTM1 were calculated in *NUPR1* KD or scrambled Cal27 and HN6 cells. At least 30 cells were chosen to calculate the results. Scale bar = 10 μm; n=3; $^{**}P<0.01$ vs. the scrambled group; $^{\#\#}P<0.01$ vs. the *NUPR1* KD group.

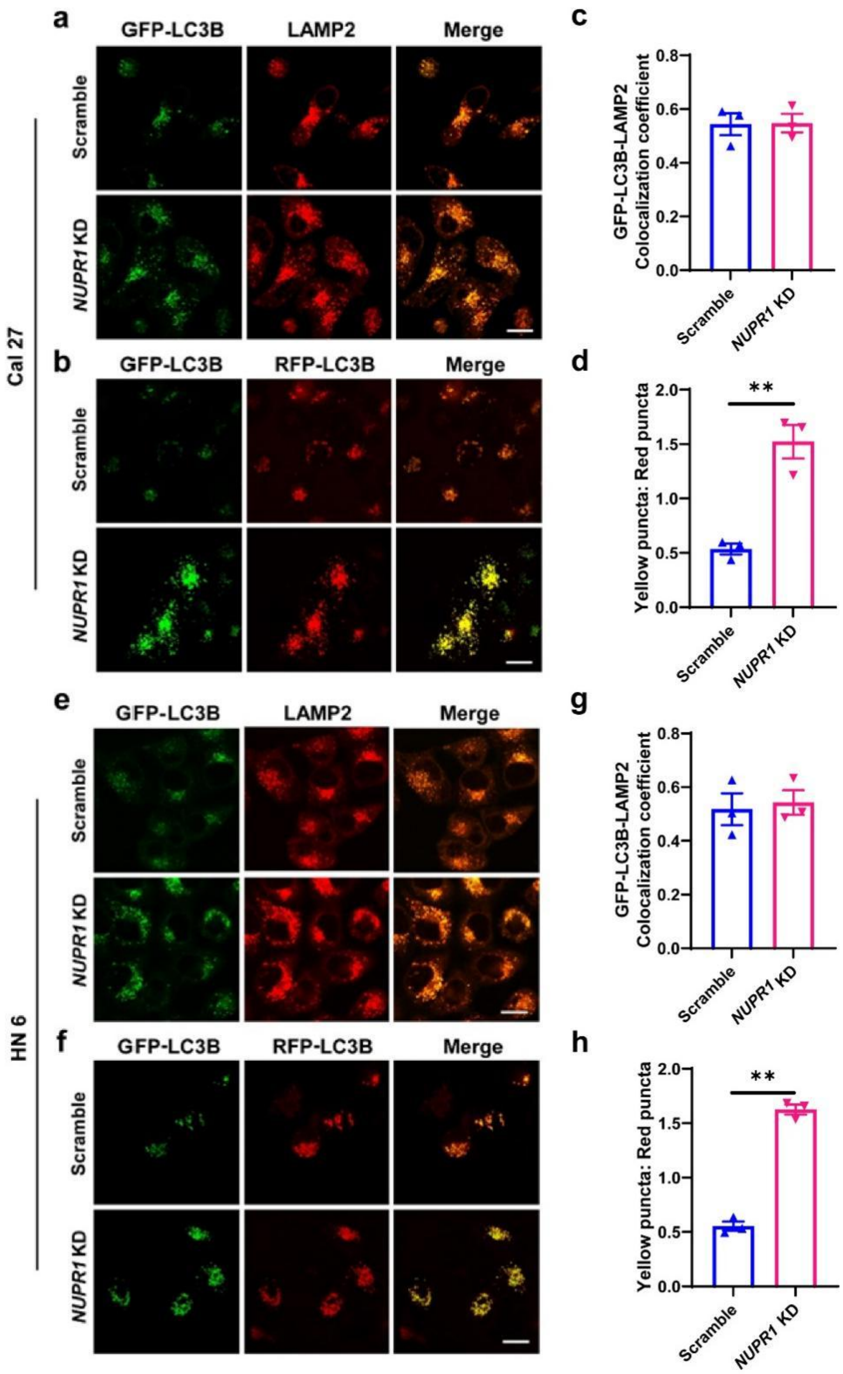

Figure. S5. *NUPR1* KD did not disturb autophagosome-lysosome fusion in OSCC cells. **a.c.e.g** Representative immunofluorescence images and colocalization coefficient analysis of GFP-LC3B puncta and LAMP2 puncta in *NUPR1* KD or scrambled Cal27 and HN6 cells. At least 30 cells were chosen to calculate the results; n=3; Scale bar: 10 μm. **b.d.f.h** Representative immunofluorescence images and the ratio of yellow puncta and red puncta analysis in *NUPR1* KD or scrambled Cal27 and HN6 cells following transfection with RFP-GFP-LC3B. At least 30 cells were used to calculate the results; n=3; Scale bar: 10 μm. ***P*<0.01 vs. the scrambled group.

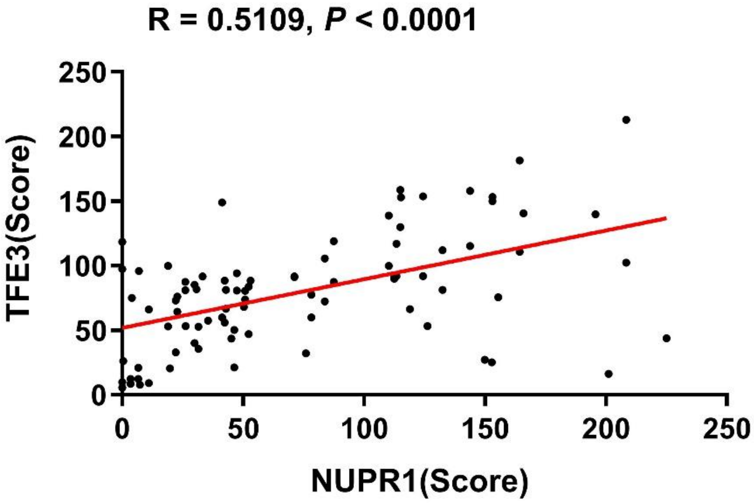

Figure. S6. A positive correlation between NUPR1 and TFE3 in OSCC tissues.n=88.

Table S1. Differentially expressed proteins in LNM compared with non-LNM (TOP 5 increase)

UniProtKB ID	Protein name	Protein description	Fold change
Q969P6	MUC5B	Mucin-5B OS=Homo sapiens OX=9606 GN=MUC5B PE=1 SV=3	1895.998
Q8WXE9	BPIFB2	BPI fold-containing family B member 2 OS=Homo sapiens OX=9606 GN=BPIFB2 PE=1 SV=2	76.556
O60356	NUPR1	Nuclear protein 1 OS=Homo sapiens OX=9606 GN=NUPR1 PE=1 SV=2	14.077
P49674	PLAA	Phospholipase A-2-activating protein OS=Homo sapiens OX=9606 GN=PLAA PE=1 SV=2	12.631
Q96ME7	RPTN	Repetin OS=Homo sapiens OX=9606 GN=RPTN PE=1 SV=1	7.33

Table S2. Relationship with the patient's clinicopathological characteristics and NUPR1 expression alteration in OSCC patients

**Variable**	**n**	**NUPR1 Expression level**		***P***
		**High**	**Low**	
Age				0.4034
<60 years	61	33	28	
≥60 years	27	12	15	
Gender				0.5982
Male	59	21	38	
Female	29	12	17	
Smoking				0.2882
Yes	72	30	42	
No	16	9	7	
Drinking				0.4191
Yes	34	14	20	
No	54	27	27	
Differentiation				0.000
High	62	18	44	
Moderate/low	26	20	6	
TNM stage				0.008
I/II	45	21	24	
III/IV	43	32	11	
Lymphatic metastasis				0.000
N0	41	23	28	
N+	47	41	6	

Table S3. Number of differentially expressed proteins between *NUPR1* KD and scramble control

Filtering Criteria	Fold change >1.2 or < 0.8 and P value < 0.05		
Comparisons	Significantly changing		
	Upregulated	Downregulated	All
NUPR1 KD_vs_Scramble	27	28 (including TFE3)	55

Table S4. Antibodies used for the western blot experiments.

Antigen	Dilution	Catalogue number	Supplier
MAP1LC3B	1:1000	L7543	Sigma
GAPDH	1:5000	G9545	Sigma
ACTB	1:5000	A1978	Sigma
P62/SQSTM1	1:1000	Ab91526	Abcam
TFE3	1:1000	PA5-99215	Invitrogen
LAMP1	1:1000	50-1071-82	Invitrogen
LAMP2	1:1000	MA1-205	Invitrogen
NUPR1	1:1000	PA1-4177	Invitrogen
anti-mouse (secondary antibody)	1:1000	A0208	Beyotime Company
anti-rabbit (secondary antibody)	1:1000	A0216	Beyotime Company

Table S5. Antibodies used for the immunofluorescence experiments.

Antigen	Dilution	Catalog number	Supplier
SQSTM1	1:100	ab56416	Abcam
LAMP2	1:100	ab13524	Abcam
Alexa Fluor® 647 goat anti-rat IgG (H+L) antibody	1:200	A21247	Invitrogen
Alexa Fluor® 568 donkey anti-mouse IgG (H+L) antibody	1:200	A10037	Invitrogen

Table S6. Sequences of primers used in quantitative RT-PCR

Target gene	Primer	Nucleotide sequence
NUPR1	F	5'- TCAACAGATGTCGGGGGAGA-3'
	R	5'- TCTGCAGTGTGGGGCTTATG-3'
VPS16	F	5'-TACACGGCGAACTGGAACC-3'
	R	5'-GCCTCACACTAGCAGCTTTCT-3'
ATG7	F	5'- CATCCGCGTGACCGTCTAA-3'
	R	5- GGGTGGTGTACTGAGGTGTT-3'
ATP6V0D1	F	5'-TTCCCGGAGCTTTACTTTAACG-3'
	R	5'-CAAGTCCTCTAGCGTCTCGC-3'
CLCN7	F	5'-CCCACACAACGAGAAGCTCC-3'
	R	5'-ACTTGTCGATATTGCCCTTGATG-3'
CTSB	F	5'-GAGCTGGTCAACTATGTCAACA-3'
	R	5'-GCTCATGTCCACGTTGTAGAAGT-3'
LAMP-1	F	5'-TCTCAGTGAACTACGACACCA-3'
	R	5'-AGTGTATGTCCTCTTCCAAAAGC-3'
TRPM1	F	5'-GTTCACCAACCAGCATATCCC-3'
	R	5'-GCTTTATTGGAATATCCGCCACC-3'
TFE3	F	5'-CCGTGTTCGTGCTGTTGGA-3'
	R	5'-GCTCGTAGAAGCTGTCAGGAT-3'
ACTB	F	5'-CATGTACGTTGCTATCCAGGC-3'
	R	5'-CTCCTTAATGTCACGCACGAT-3'
